# The Multi‐Functional Use of Large Tree Cavities by Arboreal Vertebrates in a Temperate Broadleaved Forest of Eastern Europe

**DOI:** 10.1002/ece3.70521

**Published:** 2024-11-07

**Authors:** Yehor Yatsiuk

**Affiliations:** ^1^ Institute of Ecology and Earth Sciences University of Tartu Tartu Estonia

**Keywords:** cavity nester, keystone structure, motion‐activated camera, species interactions, tree hollow

## Abstract

Tree cavities offer protected shelters and resources for arboreal vertebrates worldwide. In general, cavities with larger openings are better accessible for predators and are avoided by smaller species for breeding, but can still be attractive for occasional use. The current study explores the diversity of functional use types and species interactions at the largest available tree cavities (entrance width ≥ 10 cm) in a temperate European forest with a low number of large cavity‐breeding species. Year‐round camera observations at 9 cavities (range 0.7–3.5 years) revealed 34 visiting species of birds and mammals, including non‐cavity‐breeding species. The top predator threatening other large‐cavity users was European pine marten (*Martes martes*), which regularly visited each cavity year‐round, on average every 0.7 months. Tawny owl (*Strix aluco*) was the only species successfully breeding in cavities, arguably because of its ability to defend the nests. However, other species visited cavities at an average rate of 1.5 visits per day, making predominantly short visits (less than 30 s) interpreted as exploration, searching for food, or inspecting for the presence of owls (mobbing). Making short visits and time segregation with predators was a behavioural strategy to exploit cavities for most species. These results confirm that, similarly to other keystone structures (large arboreal nests, ground burrows, etc.), large tree cavities attract a significant part of the arboreal vertebrate community and enrich their habitats. To sustain these functions in wooded ecosystems, management should provide a surplus of available cavities and diversity of their characteristics even when the apparent number of breeding species is low.

## Graphical abstract

Year‐round camera observations at cavity entrances in a temperate forest revealed their visitation by 34 bird and mammal species. Prolonged use for breeding and roosting was constrained by regular visitation of a pine marten (*Martes martes*). Instead, most species, including non‐cavity‐nesting birds, made short visits associated with resource acquisition and social interactions. It highlights the role of large cavities in enriching the environment of arboreal vertebrates.

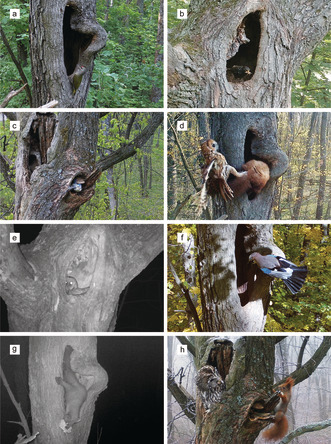

## 1 Introduction

Tree cavities are microhabitats with enclosed space and openings, formed by fungal decay processes, animal excavation, or a combination of these processes in trees. For vertebrate animals, cavities can provide shelters from predators and stable microclimate conditions, offering breeding, resting, feeding, or watering opportunities (Gibbons and Lindenmayer 2002; Wesołowski and Martin 2018; Patel, Sivaraman, and Balakrishanan 2021). These functions appear worldwide: many arboreal species are dependent on the availability of suitable cavities or use these facultatively (Goldingay 2011, 2009; Renton et al. 2015; Rhodes, O'Donnell, and Jamieson 2009; van der Hoek, Gaona, and Martin 2017). Diverse roles of cavities for arboreal vertebrates highlight their role as ‘keystone structures’ (Tews et al. 2004). Characteristics of cavity supply are proposed to be used as indicators of habitat values (Asbeck et al. 2021; Le Roux et al. 2014; Paillet et al. 2018). Best practices to address higher cavity availability in landscapes include protecting existing cavity trees and ensuring natural formation of cavities (Ball, Lindenmayer, and Possingham 1999; Gibbons and Lindenmayer 1996; Manning et al. 2013), protecting cavity excavators (van der Hoek et al. 2020), and creating artificial cavities (Ellis, Taylor, and Rhind 2022; Rueegger 2017). In order to better address the requirements of the arboreal animal communities, these approaches should also consider regional sets of cavity users and their specific requirements.

Provision of safe breeding sites is perhaps the best‐known function of cavities for vertebrates. The suitability of breeding cavities for particular species is primarily determined by cavities' dimensions, their accessibility for local predators, and competition with other species. The general tendency is that larger animal species use larger cavities (Goldingay 2009; Renton et al. 2015). Under available predation pressure, animals choose entrance dimensions closer to their body size or defend their nests to prevent the access of larger predators (Wesołowski 2007; Goldingay 2011; Wesołowski and Martin 2018; but see Rhodes, O'Donnell, and Jamieson 2009 for the case of lower predation pressure). As direct competition is difficult to observe, it is usually studied by comparing preferred cavity characteristics and time of use between potential competitor species (Cornelius et al. 2008; Renton et al. 2015). Such studies show that interspecific competition may lead to time segregation in the use of similar cavities (Brightsmith 2005; Garnett, Pedler, and Crowley 1999; Zahner, Bauer, and Kaphegyi 2017) or the use of different shelter types (McComb and Noble 1981; Prince 1968; Remm, Lõhmus, and Rosenvald 2008; Renton and Brightsmith 2009). Consequently, numerous studies showed different sets of breeding species in relation to cavity size (Martin, Aitken, and Wiebe 2004; Goldingay 2009, 2011; Di Sallo and Cockle 2022).

Non‐breeding uses of cavities include denning or roosting inside (e.g., Brainerd et al. 1995; Bull, Akenson, and Henjum 2000; Isaac, De Gabriel, and Goodman 2008; Yatsiuk and Wesołowski 2020), feeding (Kobayashi et al. 2022), caching food (Kobayashi et al. 2022; Masoero et al. 2018; Vigliotti 2020), and drinking or bathing in water‐filled cavities (Delgado‐Martínez, Cudney‐Valenzuela, and Mendoza 2021; Gossner et al. 2020; Sharma et al. 2016; Kirsch et al. 2021; Vickers, Hunter, and Hawes 2014). Cavities may be used for courtship displays, in some cases having distinct characteristics from those used for breeding (e.g., for Palm cockatoos *Probosciger aterrimus*: Heinsohn et al. 2017). While such use types may not be as critical in species' life cycles as the opportunity to reproduce, the presence of such cavities may affect animal habitat quality through multiple indirect and cumulative effects and mediate the spread of parasites and microorganisms (Elliott et al. 2019). Obtaining quantitative descriptions of non‐breeding use is more challenging as animals are not present in cavities as long as when breeding and leave fewer traces. Remote cameras are well suited for recording such events and have already been successfully used to address similar problems (Moore et al. 2021).

Relatively larger tree cavities can have distinct patterns of non‐breeding use, as they are more prominent, longer‐living (Kõrkjas, Remm, and Lõhmus 2021), physically accessible for a larger number of species, and host richer invertebrate communities (Henneberg et al. 2021; Micó 2018). Thus, comparative studies using remote cameras already showed higher visitation rates of large cavities compared with small ones (Kobayashi et al. 2022; Penton et al. 2021). On the other hand, larger cavities require larger trees and a longer time to form and tend to be less abundant than small cavities, especially in production forests (Fox, Hamilton, and Occhipinti 2009; Jensen, Kabrick, and Zenner 2002; Remm, Lõhmus, and Remm 2006). It may contribute to increased visitation and competition for larger cavities. Observations in different systems may show the functions provided by large cavities for communities of cavity‐using species.

This study is aimed to explore the breeding and non‐breeding use of large tree cavities in a European temperate broadleaved forest with a low diversity of large cavity‐breeding species. According to the published classification of tree‐related microhabitats (Kraus et al. 2016) or similarly to size classes reported in other studies (e.g., Gysel 1961; Wormington and Lamb 1999; Fox, Hamilton, and Occhipinti 2009), large cavities are defined here as having an entrance width equal to or larger than 10 cm. Compared with most of temperate Europe, the study area in East Ukraine lacks cavity‐nesting Stock doves (*Columba oenas*), ducks, and most owl species except Tawny owls (*Strix aluco*) (Keller et al. 2020). Black woodpecker (*Dryocopus martius*), excavating cavities with up to 8–10 cm entrance width (Glutz von Blotzheim, Bauer, and Bezzel 1980), is present in the study area in low numbers (Yatsiuk and Viter 2024), and cavities produced by this species were not studied. Previous control of all known large cavities in the study plot suggests that only the Tawny owl regularly bred in the observed cavities, with accidental findings of two breeding mammal species (Forest dormouse *Dryomys nitedula* and Eurasian red squirrel *Sciurus vulgaris*) and Hornet wasp (*Vespa crabro*) colonies (Y. Yatsiuk, unpublished data). No data were available on non‐breeding use of large cavities before the study.

It is expected that accessibility of large cavities for predators leads to their low use as breeding sites and shelters, but they remain attractive for other use types such as feeding sites. The specific questions asked are: (i) what part of the local arboreal community is visiting large cavities and for which function; (ii) what is the role of predation risk in the use of large cavities; (iii) what might be the role of large cavities as sites for social interactions in the vertebrate community; and (iv) what is the level of possible competition for cavities as breeding sites.

## 2 Methods

### 2.1 Study Area

**FIGURE 1:**
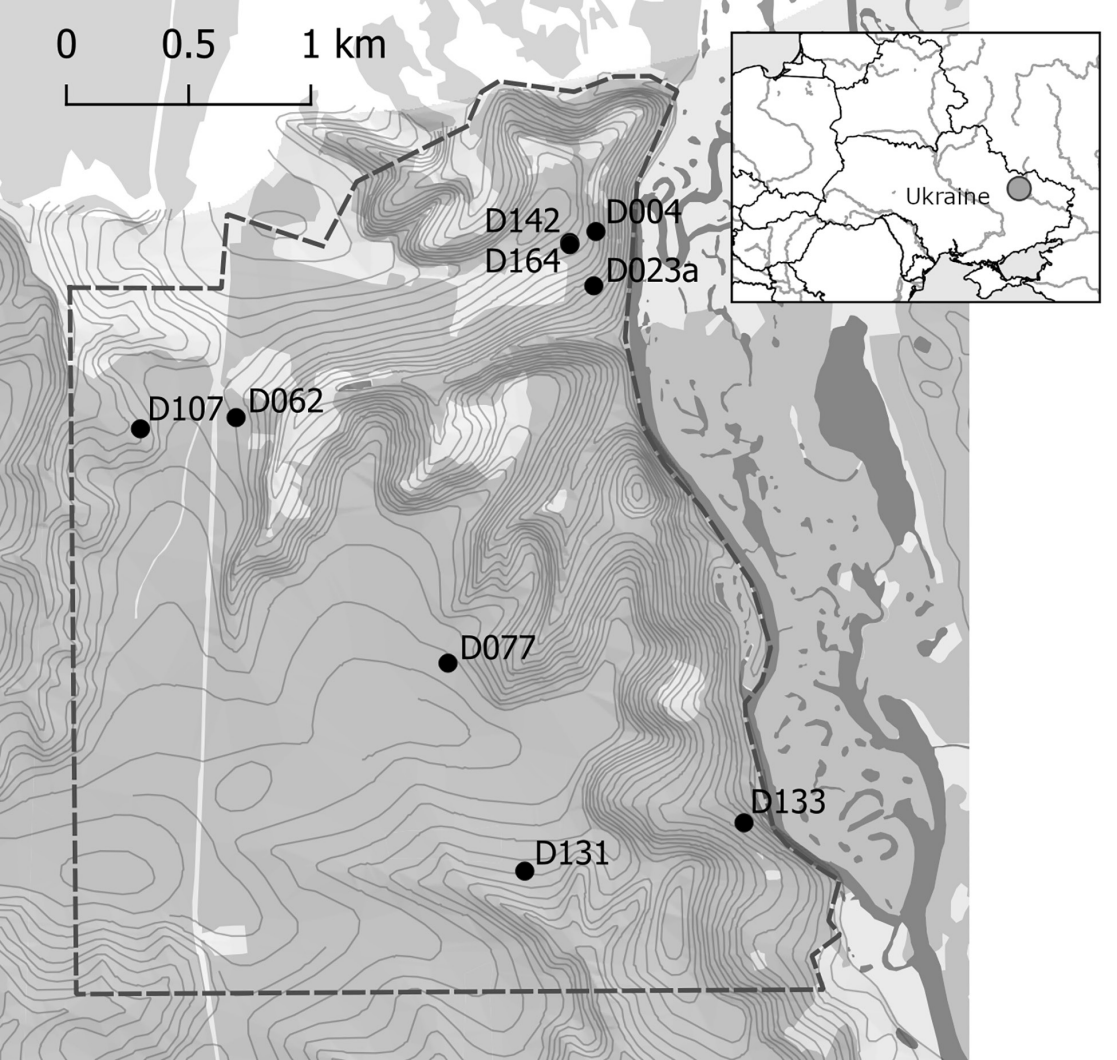
Location of the study region and the camera‐equipped cavities within the study plot. The map inset shows the location of the study plot on a regional level. On the main map, forests are shown in grey, and observed cavities are represented by dots with numbers referred to in the text; other known cavities are not shown.

The study was conducted in a broadleaved oak‐dominated forest in Homilsha Forest National Nature Park (Kharkiv region, Ukraine: 49°36′ N, 36°19′ E, Figure 1). This area is located in a transition zone between East European forest‐steppe and Pontic steppe ecoregions (Olson et al. 2001). The mean air temperatures are between +21°C in July and −4.6°C in January with average annual precipitation 527 mm (1990–2019 norms for Kharkiv; Zepner et al. 2021). Average duration of snow cover is 90–100 days, between December and end‐March but with frequent thawing. On the 750‐ha study plot, due to the establishment of a protection regime, production silviculture had been ceased since 2007, but during the study period, the conditions and availability of tree cavities were still close to mature managed stands in the region. Mean stand ages were between 90 and 120 years, typical for mature broadleaved forests in the region.

### 2.2 Cavity Selection

The information about tree cavities available on the plot was obtained from a previous study focused on the supply of potential breeding and roosting cavities used by Tawny owls (Yatsiuk and Wesołowski 2020). Of the 63 large cavities known on a study plot, nine were selected for observations. All were decay‐formed trunk cavities with entrance width ≥ 10 cm, at 3.6–12 m height, and a flat base filled with a mixture of decaying wood and other organic remains (wood mould). These cavity characteristics correspond to microhabitat type CV23 as defined by Kraus et al. (2016) and represent the largest size class of cavities available within the plot. The mean distance between cavities was 428 m; however, four cavities were clustered in the northern part of the plot with mean distance of 83 m due to the distribution of mature stands (Figure 1). Detailed descriptions of cavities and trees are provided in Table A1: Appendix.

### 2.3 Camera Setting

The camera observations were made between January 2018 and November 2023. Four models of cameras were used in this study: Bushnell Trophy Cam HD Aggressor No Glow 2017 (3 cameras), Browning BTC‐8FHD (2 cameras), Browning BTC‐6HD‐940 (2 cameras), and Stealth Cam G34 (3 cameras). The first three models had IR flash, and the last one had a low‐glow red flash. Cameras were rotated between cavities during the study with an average 1‐year period.

Trees were accessed using single‐rope technique, and cameras were attached to branches by straps and ropes in front of cavities or to neighbouring trees 2–5 m away, so that cavity entrances and their closest surroundings could be visible. Cameras were set to still mode, making 3 captures per trigger with the delay time of 10 s, trigger sensitivity was set to the highest. Cameras were checked on average every 2–3 months, but check intervals were increased up to 10–12 months in the last 2 years of the study; the maximal term when the camera still worked between two consecutive checks before draining batteries or filling memory was 10.8 months.

### 2.4 Measurements

The unit of observation was a visit by a vertebrate. Preliminary exploration of data showed that the duration of 91% of visits associated with exploration, feeding, or social interactions were equal or lasted less than 30 s. In order to capture such events and account for possibly different individuals visiting cavities, the interval between images indicating different visits was taken as 30 s. When animals used cavities for roosting, a ‘visit’ was one roosting event per day, irrespective of its length. If two or more species were observed within one event, they were treated as one visit for each species.

The information recorded for each visit was: animal species, number of individuals, time and date of observation, visit duration, activity type. Activity types were classified into five categories: passing with no interest to the cavity (excluded from analysis), approaching and looking inside, entering the cavity, roosting in cavities, and breeding in cavities. The first four categories refer to non‐breeding use. Detailed descriptions of activity types and associated observations in camera images are provided in Table 1: they are listed in the order of increasing importance in the animal life cycle and, in parallel, increasing animal vulnerability for predation in cavities.

**TABLE 1:** Description of cavity use types by vertebrates discussed in the current study and their classification on camera images. The types are arranged in an increasing order of importance for animal life cycle, visit duration, and vulnerability for predation. The first four types represent non‐breeding use of tree cavities, as discussed in the introduction.

	Point of interest	Exploration and obtaining resources	Opportunistic shelter	Roosting or denning shelter	Breeding shelter
Behaviour observed on camera images	Look from the distance or approach; look inside the cavity: short visits	Enter cavities Feeding or other behaviour: short visits	Enter the cavities and spend a long time inside, but occasional use, mainly during animal activity hours	Enter cavity and spend long time inside. Regular use, outside activity hours (e.g., at night for diurnal species)	Building nests, regular visits by pairs or females in a breeding context, appearance of young animals
Interpreted behaviour	Explorative behaviour	Explorative behaviour, obtaining resources	Escape from predators or bad weather Short‐term rest	Sleeping or hibernating inside (spend vulnerable periods)	Breeding inside
Vulnerability for predation and importance in a life cycle	Low	Low or medium	Medium	High	High or critical

When possible, feeding, displaying, and other behaviour was additionally recorded for each visit. Feeding was stated when animals were seen with food items or by characteristic behaviour (inspecting cavity walls, pecking, martens predating bird nests, etc.). Interactions were recorded when more than one individual was seen during the same visit (series of photos) or when animals visited active nests with adult birds or chicks inside. Interactions were categorised as predation, aggression, mobbing of owls, and neutral. Visit duration was calculated as the time between the first and last image for each visit (given the delay time between triggers, the shortest recordable visit duration was 10 s). Visitation rates were calculated for each species for each cavity/month series of observations by dividing the number of visits by the number of days when cameras operated at each cavity. Although infrared cameras were not intended to document visits of invertebrates, activity of Hornet wasps at cavities was frequently recorded, especially at wasp colonies.

For each visit, it was identified whether it occurred during or outside the time when the cavity was used for breeding by any vertebrate. As exact laying dates were not possible to estimate, the onset of breeding was established by the beginning of everyday visits by the breeding animals (usually multiple visits a day), and the end of breeding was set after the last image of juveniles or adults made in two consecutive days.

To compare proportions of cavity‐visiting species with their share in local breeding assemblages, the data of breeding bird counts in oak forests of the National Park were assembled based on publications (Vergeles, Gorelova, and Drulyova 1994) and the Nature Record of the National Nature Park Homilsha forest (censuses in 2007–2010). The breeding bird census data were averaged between years, and the relative proportion of each species was calculated. These data were compared with the proportion of visits for each species made between April and September, when most migrating species are still present in the area. Cavity‐nester status for each bird species was taken from a published review (van der Hoek, Gaona, and Martin 2017).

### 2.5 Data Analysis

Image processing, including assigning tags indicating animal species, number of specimens, and activity types, was done using DigiKam software (www.digikam.org). All statistical calculations were made in R version 4.3.2 (R Core Team 2023). Image metadata with tag information was extracted from images and grouped into events with 30‐s lag using R package ‘camtrapR’ (Niedballa et al. 2016).

Overlaps in diel visitation activity were estimated between short visits of predatory species (Tawny owl and European pine marten) and potential prey (small diurnal birds, terrestrial rodents, squirrels, and bats). Small diurnal birds included woodpeckers and passerine species; terrestrial rodents included *Apodemus* mice and bank voles (*Myodes glareolus*). All visits of martens were used in calculations, but for Tawny owls only short visits were used, assuming that the presence of roosting or nesting owls in cavities will influence the activity of other species. Time was transformed to local solar time (Vazquez et al. 2019) using the ‘Activity’ package (Rowcliffe et al. 2014). Temporal segregation in visitation activity was tested for each season using non‐parametric kernel density functions to calculate activity overlap coefficients ranging between 0 (no overlap) and 1 (complete overlap). The 95% confidence intervals of overlap coefficients were obtained through 1000 bootstrap samples. Activity overlap index Δ1 was used, as seasonal numbers of marten visits were between 19 and 41, and calculated using the ‘Overlap’ package (Ridout and Linkie 2009). To test for homogeneity of tested samples, Mardia–Watson–Wheeler test was performed using the ‘Circular’ package (Agostinelli and Lund 2017).

## 3 Results

### 3.1 Use of Cavities by Local Vertebrate Community

In total, the observations at nine cavities covered 6279 trap nights (Table A1: Appendix). Throughout this time, 15,191 visits of 34 vertebrate animal species (29 birds and at least 5 mammals with bats and small rodents not identified to species in most cases) were recorded (Table 2). Birds ranged in size from small passerines (European treecreeper *Certhia familiaris*, Wood warbler *Phylloscopus sibilatrix*) to medium‐sized species (European honeybuzzard *Pernis apivorus*, Tawny owl, Common raven *Corvus corax*). Mammals comprised four species of rodents, including terrestrial wood mice (*Apodemus* sp.) and bank vole (*Myodes glareolus*), mostly unidentified bats (only long‐eared bats *Plecotus* spp. could be identified), and European pine marten. The most active visitor was the Tawny owl (59.8% of all visits), followed by terrestrial rodents, the Eurasian red squirrel, and a set of cavity‐nesting bird species, but also non‐cavity‐nesting birds (Table 2).

**TABLE 2:** Summary of vertebrate species visiting 9 large cavities in a broadleaved forest in East Ukraine recorded year‐round remote camera observations (6279 trap nights totally). Use types are stated according to descriptions in Table 1.

Common name	Scientific name	Point of interest, *n* visits^a^		Exploration and obtaining resources, *n* visits^b^		Opportunistic shelter, *n* visits	Roosting or denning, *n* visits	Breeding shelter, *n* cases^c^
Birds								
Tawny owl	*Strix aluco*	376		3442	FC		299	4 (3)
Eurasian nuthatch	*Sitta europaea*	369	MM	424	F			
Great tit	*Parus major*	273	MM	357	F			
Middle spotted woodpecker	*Dendrocoptes medius*	145	M	143	F			
Grey‐headed woodpecker	*Picus canus*	135	M	75	F			
Collared flycatcher	*Ficedula albicollis*	126	M	50	F			
Eurasian blue tit	*Cyanistes caeruleus*	65	MM	53	F			
Eurasian treecreeper	*Certhia familiaris*	60	M	40	F			
Eurasian wryneck	*Jynx torquilla*	39	M	57				1 (0)
Great spotted woodpecker	*Dendrocopos major*	65	M	6	F			
Eurasian jay	*Garrulus glandarius*	65	MM					
Eurasian blackbird	*Turdus merula*	32	MM	24	F			
Black woodpecker	*Dryocopus martius*	39		6	F		2	
Common chaffinch	*Fringilla coelebs*	26	M		F			
European robin	*Erithacus rubecula*	4	M	9	F			
Marsh tit	*Poecile palustris*	9	M	2	F			
Mistle thrush	*Turdus viscivorus*	10	M		F			
Common starling	*Sturnus vulgaris*	5	M	1	F			
Common raven	*Corvus corax*	4						
Song thrush	*Turdus philomelos*	3	M					
Hawfinch	*Coccothraustes coccothraustes*	1	M	1				
Lesser spotted woodpecker	*Dryobates minor*	2			F			
Spotted flycatcher	*Muscicapa striata*	2						
Fieldfare	*Turdus pilaris*	2			F			
European goldfinch	*Carduelis carduelis*	1						
Red‐breasted flycatcher	*Ficedula parva*	1						
European honey‐buzzard	*Pernis apivorus*	1			?F			
Common redstart	*Phoenicurus phoenicurus*	1						
Wood warbler	*Phylloscopus sibilatrix*	1	M					
Mammals								
Terrestrial rodents	*Apodemus* sp., *Myodes glareolus*	3		1452	F			
Eurasian red squirrel	*Sciurus vulgaris*	519	MM	510	F	31		
Forest dormouse	*Dryomys nitedula*	4		263	F			
European pine marten	*Martes martes*	11		101	F	1		
Bats	Chiroptera	87		21				
Sum of events		2486		7037		32	301	5 (3)
Number of species		34 (20 M)		21 (21 F)		2	2	2 (1)

^a^ Species involved in interactions with Tawny owls using cavities (mobbing) are marked: M—rare events (< 10 total occurrences), MM—numerous events.

^b^ F—feeding inside cavities registered; ?F—visit of active Hornet wasp nest but no feeding recorded; FC—Food caching in cavities.

^c^ Number of successful breeding attempts is given in parentheses.

During the observation period, one cavity tree fell and one cavity lost its base due to partial destruction of a tree trunk below the cavity. No cavities appeared water‐filled during the study, but in one case a cavity was completely filled with snow for 2 months in late winter (late January to late March, 2018), preventing visitation of animals.

**FIGURE 2:**
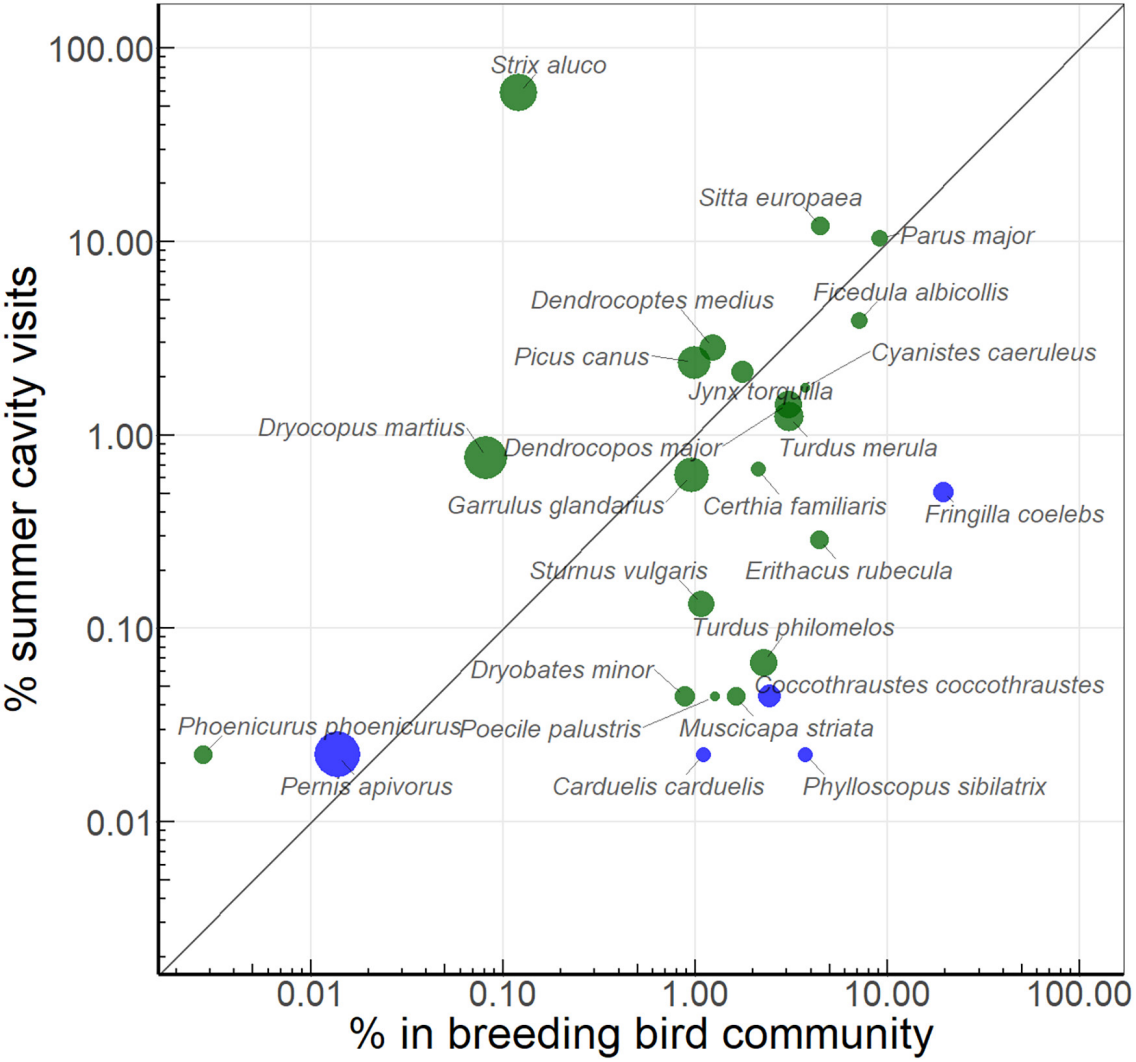
Relative visitation frequency of 25 breeding bird species in April–September comparing with their share in the local breeding community: Upper left position indicates relatively higher use of the cavities. Dot size is proportional to bird body length, and colour indicates its cavity nester status (van der Hoek, Gaona, and Martin 2017): Green—obligate and facultative cavity nesters; blue—not cavity‐nesters. Note logarithmic scales on both axes.

Of 64 bird species recorded in the oak forest interior in the study area, 27 (42%) were registered visiting cavities, including six obligate and 15 facultative cavity‐nesters, and 6 species for which cavity‐nesting was never recorded (according to supplementary data from van der Hoek, Gaona, and Martin 2017). Only two cavity‐nester species present in the local community were not registered at the cavities: the Eurasian hoopoe (*Upupa epops*), which breeds only at forest edges, and the European pied flycatcher (*Ficedula hypoleuca*), which is a rare species in this habitat. Comparison of cavity visitation rates by birds in April–September with each species' share in the breeding community showed that Tawny owl was proportionally the most active visitor of cavities (Figure 2), and relatively larger species tended to visit cavities more often than smaller species.

Maximal species diversity was between mid‐spring and mid‐summer (April–July), when 20–24 species were registered monthly, mainly due to visits of small passerines and bats (Table A2: Appendix). Minimal species diversity was in winter (December and January), when only 11 species visited cavities each month and migratory or hibernating species were missing. On average for non‐breeding observations, the highest mean visitation rate was between summer and mid‐autumn (June–October: 1.0–1.5 daily visits per cavity) and the lowest was in winter (December–January: 0.3–0.5).

**FIGURE 3:**
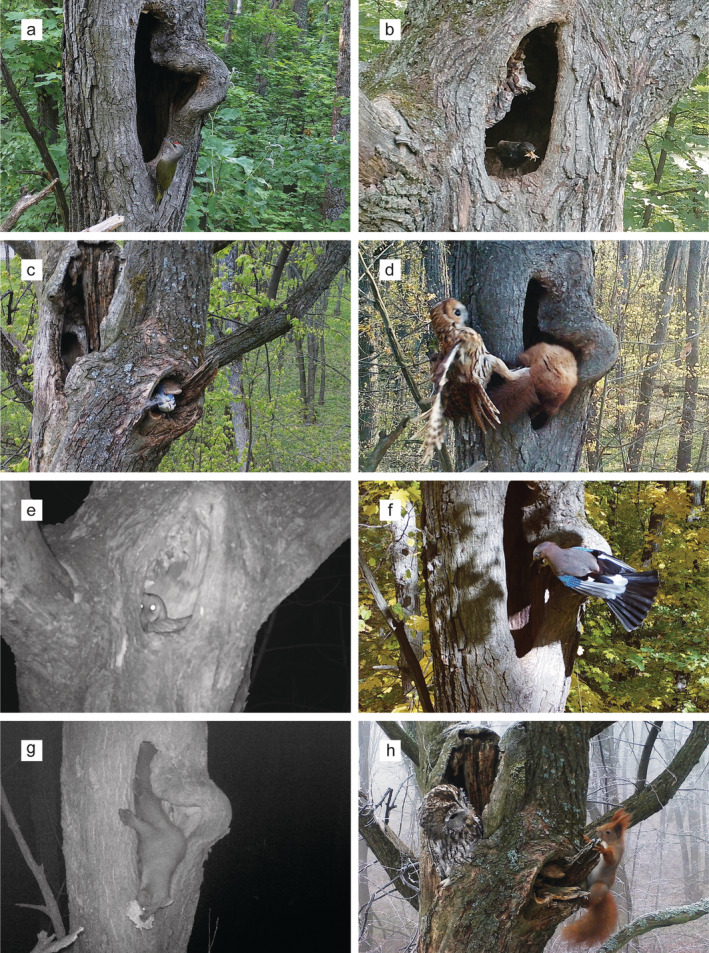
Illustrations of animal behaviour and cavity use types: (a) Grey‐headed woodpecker *Picus canus* looks inside cavity in alerted posture; (b) Eurasian blackbird *Turdus merula* feeding in cavity; (c) Eurasian blue tit *Cyanistes caeruleus* collecting nest material in active Tawny owl *Strix aluco* nest; (d) Tawny owl attacks European pine marten *Martes martes* visiting cavity with owl prey cache; (e) Tawny owl inspecting shallow cavity, apparently unsuitable for roosting; (f) Eurasian jay *Garrulus glandarius* visiting cavity previously used by roosting Tawny owls; (g) European pine marten feeding on Hornet wasp *Vespa crabro* remains in winter; (h) Eurasian red squirrel *Sciurus vulgaris* mobbing roosting Tawny owl.

Most visits were short: median duration was 10 s (10 s to 20.6 min for non‐breeding and non‐roosting observations). The approach to cavities without entering inside was demonstrated by all species at least once, and for 13 species it was the only type of behaviour (Table 2). In most cases, animals demonstrated alerted postures and tried to look inside, either from nearby branches or from the cavity entrance, looking both into the lower and upper parts of cavities (Figure 3a).

Fewer species (21) entered cavities, including non‐cavity‐nesting Hawfinch (*Coccothraustes coccothraustes*), facultative cavity‐nesting European robin (*Erithacus rubecula*), and Eurasian blackbird. Birds more often entered than approached cavities (Table 2). Before entering cavities, most species often spent some time at the entrance looking inside.

**FIGURE 4:**
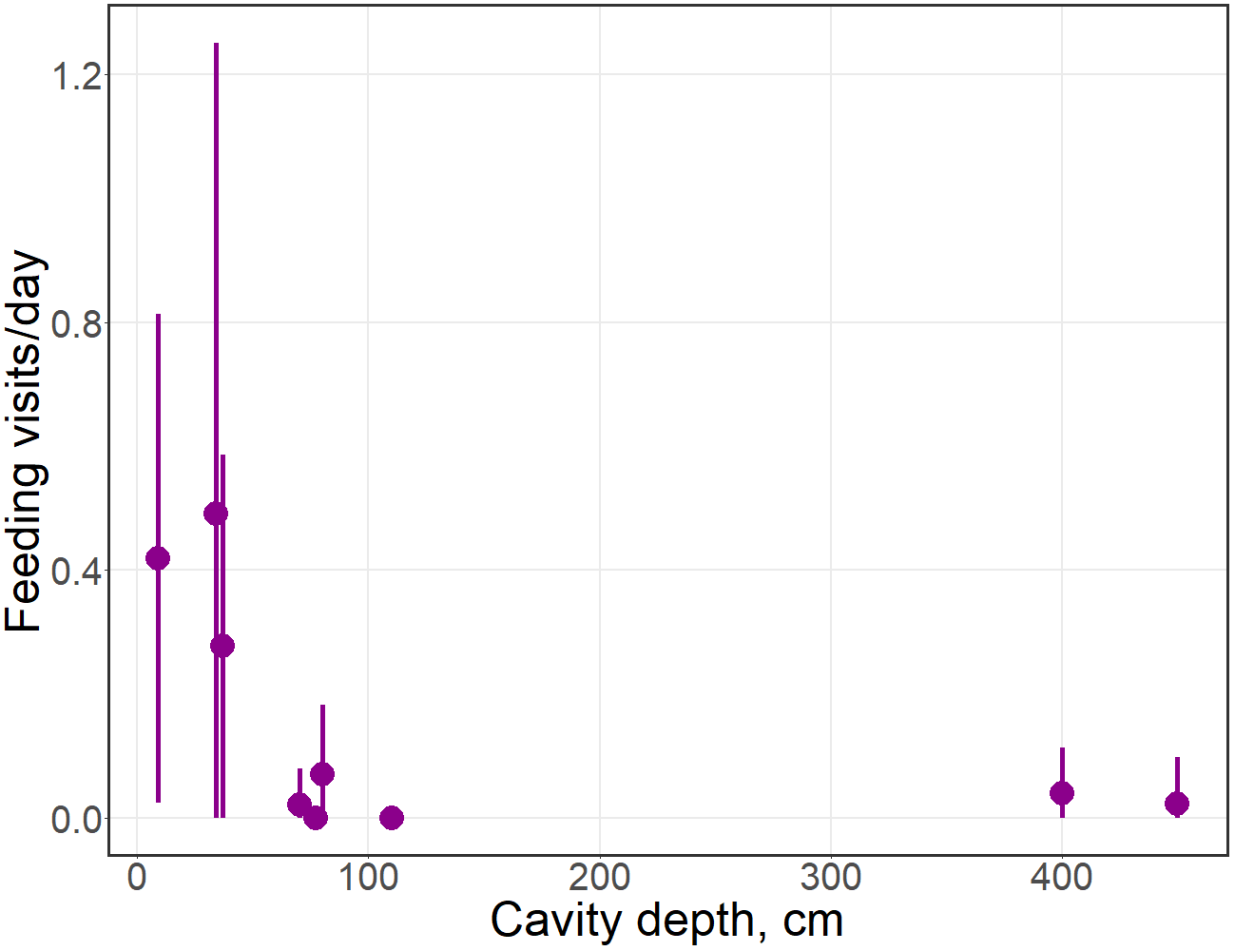
The relationship between intensity of feeding visits and cavity depth. Points indicate mean rates of feeding visits for each cavity per each month of observations, and whiskers show ±SD values.

Foraging inside cavities was registered in 10.5% of all approaching and entering visits for 20 species (Table 2) all year round. In most cases, the food items were not identified. The average frequency of feeding in shallow cavities was higher than for deep cavities (Figure 4). For example, most feeding observations of Eurasian blackbird and European robin occurred in the cavity with a 9‐cm depth (Figure 3b). The only use of water was drinking snow from cavities, mainly by wintering Mistle thrush (*Turdus viscivorus*) and Fieldfare (*T. pilaris*). Great and Eurasian blue tits were observed collecting feathers from owl nests, including visiting active nests with chicks (Figure 3c).

Tawny owls regularly made short night visits to all cavities, sometimes in pairs with peak in early spring and during the breeding season. Bats made short visits of cavities in mid‐night hours, but no roosting (regular entering in morning hours and emergence in evening hours) was observed. Only Tawny owls regularly roosted in all seasons in seven cavities. Black woodpeckers used cavities for night roosting only two times. Eurasian pine marten and Eurasian red squirrel stayed in cavities only during their activity hours, indicating the use of cavities as opportunistic shelters. Most such visits of squirrels occurred prior to breeding season (peak in February–April), with > 90% of visits lasting less than 1 h.

Only two species attempted to breed during the 22 observed breeding seasons (mid‐March—mid‐June each year per cavity): Tawny owl (4 attempts, including 1 unsuccessful) and Wryneck (*Jynx torquilla*, an unsuccessful attempt) (Table 2). Both unsuccessful breeding attempts were caused by marten depredation of clutches.

### 3.2 Predation Risk

Two of the visiting species are predators of small vertebrates: Tawny owl and European pine marten (also predating the owl nests). Martens visited all cavities equally in all seasons, with a mean frequency of 1.5 visits per month (between 1 visit per 2 weeks and 1 visit per year in different cavities). Most visits were in late evening (peak between 21:00 and 23:00) or early morning (06:00–08:00) at dark. Feeding was unequivocally observed in 26.3% of events (30 visits), but martens tended to make more visits within one night after successful feeding (average 2.5 visits/night when feeding was observed versus 1.3 visits/night when no feeding was observed). Recognised feeding objects were Hornet wasp nest remains in autumn and winter, four cases of visiting Tawny owl food cache with rodent prey (Figure 3d), and two cases of bird nest predation (see above). When predating bird nests, martens made several visits during one night with 1‐min to 3‐h intervals, but no increase in visitation rate in subsequent nights after successful feeding was observed (Mann Whitney U test for inter‐visit intervals > 12 h: *Z* = 0.117, *p* = 0.91).

**FIGURE A1:**
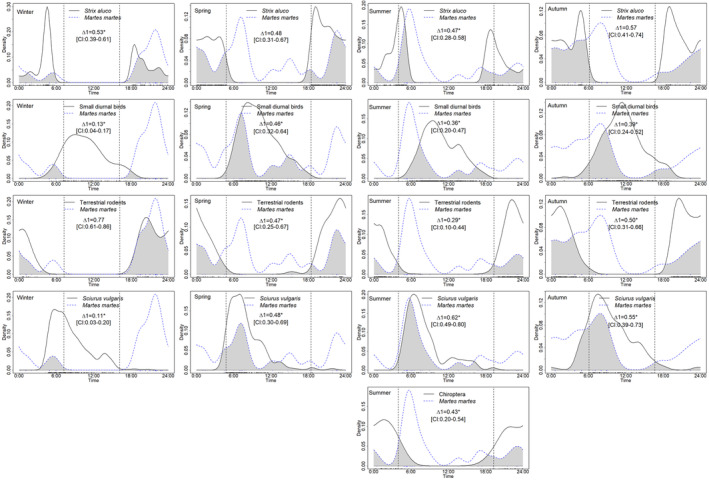
Kernel density estimates of diel activity patterns and their overlap between European pine marten and short visits of other vertebrate animal species to large tree cavities in different seasons. Overlaps in activity are shown in shaded areas. Dashed vertical lines show mean sunrise and sunset times for each season. Time is shown in solar time. Significant differences in Mardia–Watson–Wheeler are indicated by asterisks after overlap coefficient values (Δ).

Martens showed stricter nocturnal activity in winter and relatively more visits during daytime in other seasons (Figure A1: Appendix). The mean seasonal overlap in diel activity between marten and Tawny owl visits to cavities not used as breeding sites (Δ1) was 0.51 (0.47–0.53 in different seasons, see Figure A1: Appendix). Mean activity overlap with mostly nocturnal terrestrial rodents was similar (mean Δ1 = 0.51, 0.29–0.77), but lower for Eurasian red squirrel (mean Δ1 = 0.44, 0.11–0.62) bats (Δ1 = 0.43 in summer) and, especially, diurnal birds (mean Δ1 = 0.33, 0.13–0.46).

**FIGURE A2:**
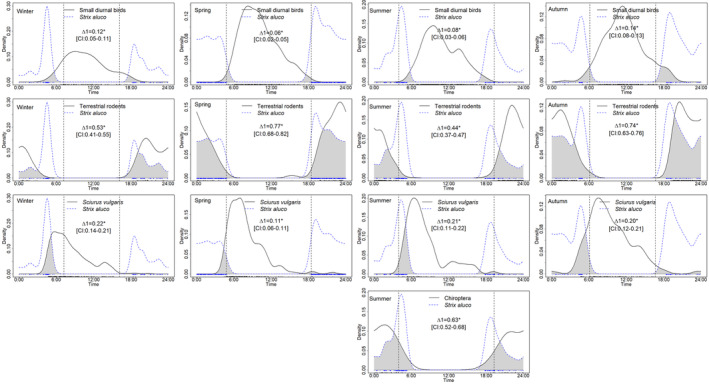
Kernel density estimates of diel activity patterns and their overlap between shirt visits of Tawny owl and other vertebrate animal species to large tree cavities in different seasons. Overlaps in activity are shown in shaded areas. Dashed vertical lines show mean sunrise and sunset times for each season. Time is shown in solar time. Significant differences in Mardia–Watson–Wheeler are indicated by asterisks after overlap coefficient values (Δ).

No catching prey by Tawny owls inside cavities was observed except for one unsuccessful attack on an Eurasian red squirrel at the cavity entrance. In two cases, roosting owls preyed in daytime hours: on Eurasian nuthatch and Bank vole. When cavities were not used for breeding and roosting, owls mainly did short visits in night time hours with peaks in late evening (18:00–21:00) and early morning (04:00–06:00). Their activity stronger overlapped with nocturnal visits of terrestrial rodents (mean Δ1 = 0.62, 0.44–0.77) and bats (Δ1 = 0.63 in summer) than with Eurasian red squirrels (mean Δ1 = 0.19, 0.11–0.22) and small diurnal birds (mean Δ1 = 0.1, 0.06–0.14) (Figure A2: Appendix).

### 3.3 Cavities as Places of Interactions

**FIGURE 5:**
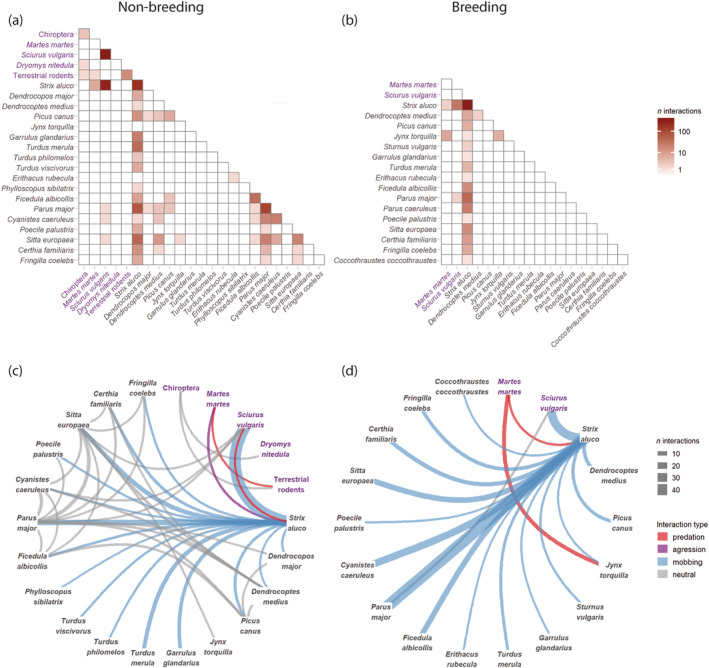
Matrices of interaction and interaction networks between vertebrate species visiting tree cavities without active nests (a, c) and with active nests (b, d). Mammal species and groups are shown in magenta, bird species are shown in dark‐grey.

In 8.3% of events, more than one species was seen at cavities (up to 4). Most interactions occurred between individuals of the same species, specifically among Tawny owls and Eurasian red squirrels (Figure 5a,b). Four types of interactions were recorded: predation (including unsuccessful attempts), aggression (Tawny owls repelling a marten), mobbing of Tawny owls at nests or roosting sites, and neutral interactions. No interactions associated with competition for cavities were observed. Both for cavities with and without active owl nests, the vast majority of interactions occurred between small birds, Eurasian red squirrels, and Tawny owls and referred to mobbing (Figures 5c,d and 3h). Visits of some species (Eurasian jay, Song thrush, *Turdus philomelos*, and Wood warbler, *Phylloscopus sibilatrix*) were only associated with mobbing owls. Neutral interactions occurred mostly between species simultaneously feeding or making short visits to cavities. Interactions occurred in all seasons, on average for all cavities 0.01–0.16 cases a day when there were no nests inside and 0.7–4.8 cases a day at active nests.

Eighteen vertebrate species visited active owl nests in March–June compared with 29 species visiting cavities without owl nests in the same season, mainly because of the absence of terrestrial rodents, forest dormouse, bats, and some bird species (Figure 5b,d). However, the remaining bird species visited active owl nests on average 3.3 more times a day than cavities with no nests at the same season.

### 3.4 Potential Competition and Overlaps in Cavity Use Terms

Tawny owl was the only vertebrate species successfully breeding in the observed cavities. Its nesting on average started on the 29th of March (05.03–08.05, *n* = 4) and ended on the 25th of June (17.05–23.08, *n* = 3, excluding one depredated nest). Except for this species, only Hornet wasp colonies occupied cavities for prolonged time (five cases of occupation by colonies in two cavities). On average, the colonies were established on the 27th of June (05.06–06.07) and lasted till October. Both mammals and birds visited cavities with wasp colonies (either looking or entering cavities), including the collection of dead insects from cavity bases. Such visits were more than four times more frequent during the first month of colony establishment, when insect aggression was lower (total 331 visit). The number of short visits by vertebrates was lower in August–September, and only in two colonies did insects constantly repel approaching animals from the beginning of a colony. Except for one late Tawny owl nest, which appeared to be replacement after predation, terms of owl nesting and establishment of Hornet wasp colonies did not overlap.

## 4 Discussion

The observations showed that, along with a single breeding species (Tawny owl), a substantial number of other vertebrate species visited the largest cavities (entrance width 10–20 cm) present in the temperate forest studied. Cavities of this size class are accessible for the vast majority of cavity‐nesting birds and mammals worldwide, except the largest cavity‐using mammals, which typically require even larger cavities (e.g., entrances wider than 30 cm for bears: Li et al. 1994; Davis 2022). It means that the observed cavity‐use patterns may be generally similar for other regions, however, involving local sets of predators and competing breeding species.

European pine marten was the key predator visiting the cavities, which apparently shaped the use of cavities by other species in the studied forest. The marten is an opportunistic predator well adapted to climbing and readily exploiting tree crown level (Jędrzejewski, Zalewski, and Jędrzejewska 1993). In contrast to other marten studies (Birks, Messenger, and Halliwell 2005; Zalewski 1997), no use of cavities for breeding and a low level of denning use were recorded in this study. However, observations showed that martens were performing regular (average interval for all cavities was 0.7 months), mostly nocturnal visits, randomly distributed across seasons. Studies on excavated cavities and nest boxes have found that predation risk for cavity‐using species increases over time, indicating that predators remember these locations (Nilsson, Johnsson, and Tjernberg 1991; Sonerud 1985). However, the lack of switching to nest box predation during low microtine population phases (Sonerud, Grønlien, and Steen 2023) implies that arboreal sites are of secondary importance for martens as foraging grounds. In the current study, the lack of increased marten visitation after successful predation suggests that prey availability in cavities is hardly predictable, and martens likely follow a random searching strategy, visiting all known cavities equally (see also Zahner, Bauer, and Kaphegyi 2017).

Such a visitation pattern indicates that prolonged use of cavities by other species can only be sustained with active defence, while short visits are possible. Tawny owl was not only the single vertebrate species successfully breeding in the large cavities, but it was also observed attacking martens. Despite such active defence (Wallin 1987), predation by martens remains one of the main causes of its nest failures (Karell et al. 2020). Overall, competition for large cavities as breeding sites was low, with only the Hornet wasps being potential competitors. Although infrared‐triggered cameras are not suited to detect insect activity, prolonged occupation by wasp colonies was clearly detectable, showing low seasonal overlap with breeding owls.

Instead of prolonged use, more than 90% of all vertebrate visits to the cavities lasted less than 30 s and were interpreted as explorative behaviour, searching for food or interactions with other visiting species. These visits were performed by the largest number of species, including those that are known to never use cavities for reproduction or as shelters. Making short visits and segregation in diel activity was apparently a way to exploit cavities but evade predation for most species. Studies in different systems have shown that, in addition to mammals, other predators like reptiles and diurnal birds of prey may frequently visit cavities, but actual predation events filmed by cameras remain rare (Cotsell and Vernes 2016; Penton et al. 2021; Schruhl et al. 2012; Zahner, Bauer, and Kaphegyi 2017). Thus, non‐breeding use associated with short visits can only be diminished under higher predation pressure: when there are more predator species with non‐overlapping activity periods or when predators are specifically focused on predation in cavities.

Searching for food was detected in most species, including not cavity‐users. However, placing cameras outside cavities did not allow to observe behaviours and food items inside cavities. Only the appearance of animals with food or typical behaviour (e.g., inspecting cavity walls by woodpeckers or other birds) was used as a sign of feeding. It may mean that the actual number of feeding events was higher, but feeding success remains unclear. Main food sources for most species in tree cavities were probably saproxylic invertebrates and colonial hymenoptera. The biomass of saproxylic communities in tree cavities is positively correlated with internal volume and cavity entrance size (Ranius 2002; Micó et al. 2015), and large cavities can provide space for larger hymenopteran colonies, which make large cavities potentially attractive feeding sites. The observations suggest that saproxylic communities in shallower cavities may face higher predation pressure due to better accessibility for birds; however, this statement needs further confirmation due to the abovementioned limitations of this study. Although cavities may generally provide few resources for local fauna, their role as additional feeding sources may be important in certain periods when the main food sources are scarce, similarly to the role of watering places in seasonally‐dry areas (Kirsch et al. 2021; Delgado‐Martínez et al. 2023) or through the provision of safer feeding opportunities for arboreal species (Sharma et al. 2016).

Most observed interactions occurred between owls and other visiting animals; they often included visible alarm displays or threatening predators and were interpreted as mobbing. The function of mobbing behaviour is often stated as informing other individuals about the presence of danger and, therefore, decreasing chances of attacks (Carlson and Griesser 2022). The recurrent mobbing events and, especially, periodic control of empty cavities by species involved in mobbing behaviour (see Figure 3f) indicated that large cavities are familiar to local fauna as sites of potential presence of predators. Mobbing behaviour is conspicuous, and, by attracting more individuals, it may further facilitate their visitation of cavities for other purposes.

The observed cavity‐use patterns are broadly similar to other permanent shelter structures used by vertebrates: large arboreal nests (Aguiar‐Silva et al. 2017; Thompson et al. 2017), ground burrows (Andersen, Bennett, and Holbrook 2021; Tye et al. 2021; Kinlaw 1999; Mukherjee et al. 2019; Linley et al. 2024), beaver lodges (Windels 2017), etc. While few species use such structures for reproduction, they can attract tens of species that make short visits and utilise such structures for resting, comfort behaviour, obtaining resources, and social interactions. All such structures are also accessible and attractive for some predators; thus, the visits are associated with increased risk of predation (Kinlaw 1999; Windels 2017), although actual predation is rarely documented (Janic et al. 2021; Thompson et al. 2017). Based on the current study and the literature, one can conclude that making short visits (Aguiar‐Silva et al. 2017) and partitioning these temporally (Tye et al. 2021; Viviano et al. 2022) or spatially (Kinlaw 1999; Mori, Menchetti, and Balestrieri 2015) are the main ways to decrease the predation risk.

In terms of management implications, the supportive role of large cavities for vertebrates described in this study can only be sustained through a surplus of cavities unoccupied by breeding animals and when the available cavities have various characteristics (dimensions, location, and mould qualities). Large cavities are formed in relatively large and old trees which tend to be rare in the landscapes (Cameron 2006; Fox, Hamilton, and Occhipinti 2009). It means that every large cavity tree present in an area may have a value for fauna even if it is unsuitable as breeding shelter. Management planning should consider retention of existing large cavity trees and ensuring the recruitment of new cavity trees in the future by retaining trees exhibiting decay progression.

## Author Contributions

**Yehor Yatsiuk:** conceptualization (lead), data curation (lead), formal analysis (lead), investigation (lead), methodology (lead), project administration (lead), resources (lead), software (lead), validation (lead), visualization (lead), writing – original draft (lead), writing – review and editing (lead).

## Conflicts of Interest

The author declares no conflicts of interest.

## Data Availability

The data that support the findings of this study are available in Dryad at https://datadryad.org/stash/share/JStkI4jdxyqdtGcXCCkmogcMR7s1vWAFNLkfFJP‐qF4.

## Appendix

**TABLE A1:** Characteristics of cavities and observation effort.

Number	Tree species	Dimensions: Tree DBH, height (entrance width × height; cavity depth)	Camera‐trapping effort: Trap days (approximate calendar term); number of breeding seasons^a^	Visitation activity: number of visiting species (number of roosting events—breeding attempts)	Cavity fate or characteristics
D004	*Quercus robur*	77 cm, 11 m (16 × 55; 70 cm)	1110 (~3 years); 3	10 (57–2)	
D023a	*Quercus robur*	72 cm, 12 m (13 × 50; 37 cm)	367 (1 year); 1	16 (0–0)	
D062	*Pyrus communis*	61 cm, 7 m (10 × 40; 110 cm)	289 (9.5 months); 1	22 (79–1)	Tree fallen^b^
D077	*Tilia cordata*	37 cm, 3.6 m (20 × 33; 300 cm)	662 (1.8 years); 2	20 (63–0)	
D107	*Pyrus communis*	50 cm, 4.5 m (16 × 24; 400 cm)	1193 (~3.5 years); 4	18 (73–0)	
D131	*Quercus robur*	47 cm, 9 m (18 × 48; 34 cm)	1008 (~2.8 years); 5	27 (29–1)	
D133	*Quercus robur*	86 cm, 11 m (20 × 41; 80 cm)	1026 (~2.9 years); 4	22 (6–0)	
D142	*Quercus robur*	53 cm, 12 m (14 × 16; 77 cm)	251 (8.4 months); 1	20 (15–1)	Partly destroyed^b^
D164	*Quercus robur*	64 cm, 9 m (15 × 20; 9 cm)	373 (~1 year); 1	18 (0–0)	Shallow cavity

^a^ Breeding season includes observations between mid‐March and mid‐June each year.

^b^ In the end of observation period.

**TABLE A2:** Mean monthly visitation rates of vertebrate species visiting 9 large cavities. in a broadleaved forest in East Ukraine recorded year‐round remote camera observations (6279 trap nights totally).

Common name	Scientific name	Visitation rate, visits/Day											
		Jan	Feb	Mar	Apr	May	Jun	Jul	Aug	Sep	Oct	Nov	Dec
Birds													
Tawny owl	*Strix aluco*	0.136	0.331	0.887	0.562	0.317	0.265	0.317	0.159	0.325	0.233	0.263	0.196
Eurasian nuthatch	*Sitta europaea*	0.038	0.074	0.070	0.041	0.089	0.306	0.215	0.135	0.159	0.175	0.083	0.066
Great tit	*Parus major*		0.007	0.098	0.106	0.108	0.218	0.107	0.038	0.171	0.123	0.024	0.006
Middle‐spotted woodpecker	*Dendrocoptes medius*	0.013	0.027	0.011	0.012	0.009	0.073	0.069	0.016	0.030	0.193	0.055	0.029
Grey‐headed woodpecker	*Picus canus*	0.015	0.011	0.004	0.005	0.006	0.022	0.073	0.028	0.051	0.073	0.065	0.036
Collared flycatcher	*Ficedula albicollis*				0.006	0.090	0.078	0.054	0.004				
Eurasian blue tit	*Cyanistes caeruleus*	0.003		0.020	0.041	0.006	0.003	0.016	0.004	0.030	0.031	0.006	
Eurasian treecreeper	*Certhia familiaris*	0.010	0.011	0.019	0.005	0.001	0.010	0.009	0.004	0.016	0.053	0.024	0.006
Eurasian wryneck	*Jynx torquilla*				0.012	0.004		0.003					
Great‐spotted woodpecker	*Dendrocopos major*	0.002		0.005	0.002	0.006	0.058	0.019	0.014	0.014	0.009		
Eurasian jay	*Garrulus glandarius*	0.005	0.014	0.014	0.008	0.011	0.005	0.011		0.008	0.024		0.012
Eurasian blackbird	*Turdus merula*				0.008	0.015	0.033	0.027					
Black woodpecker	*Dryocopus martius*		0.022		0.002	0.006	0.017	0.019	0.008	0.006	0.002		
Common chaffinch	*Fringilla coelebs*			0.004	0.008	0.003	0.007	0.003		0.004			
European robin	*Erithacus rubecula*				0.012	0.005		0.002					
Marsh tit	*Poecile palustris*			0.006							0.006	0.004	
Mistle thrush	*Turdus viscivorus*		0.004	0.001								0.010	0.008
Common starling	*Sturnus vulgaris*				0.027								
Common raven	*Corvus corax*			0.006									
Song thrush	*Turdus philomelos*					0.002		0.003					
Hawfinch	*Coccothraustes coccothraustes*				0.002								
Lesser spotted woodpecker	*Dryobates minor*							0.003					
Spotted flycatcher	*Muscicapa striata*					0.002	0.002						
Fieldfare	*Turdus pilaris*		0.004										
European goldfinch	*Carduelis carduelis*					0.001							
Red‐breasted flycatcher	*Ficedula parva*										0.002		
European honey‐buzzard	*Pernis apivorus*							0.002					
Common redstart	*Phoenicurus phoenicurus*						0.002						
Wood warbler	*Phylloscopus sibilatrix*							0.002					
Mammals													
Terrestrial rodents	*Apodemus* sp., *Myodes glareolus*	0.044	0.030	0.022	0.136	0.084	0.143	0.373	0.588	0.606	0.505	0.135	0.094
Eurasian red squirrel	*Sciurus vulgaris*	0.156	0.462	0.245	0.180	0.102	0.082	0.034	0.053	0.102	0.177	0.191	0.109
Forest dormouse	*Dryomys nitedula*				0.005	0.062	0.110	0.142	0.121	0.040			
European pine marten	*Martes martes*	0.019	0.027	0.011	0.012	0.011	0.007	0.016	0.014	0.009	0.026	0.015	0.035
Bats	Chiroptera				0.015	0.009	0.060	0.042	0.052	0.002			
Number of species		11	13	16	22	23	20	24	15	16	15	12	11
